# Impact of patient selection in clinical trials: application of ROCKET AF and ARISTOTLE criteria in GARFIELD-AF

**DOI:** 10.1136/openhrt-2024-002708

**Published:** 2024-07-01

**Authors:** Jelle C L Himmelreich, Saverio Virdone, John Camm, Karen Pieper, Ralf E Harskamp, Ali Oto, Barry F Jacobson, J P S Sawhney, Toon Wei Lim, Harry Gibbs, Shinya Goto, Sylvia Haas, Keith A A Fox, Petr Jansky, Freek Verheugt, Ajay K Kakkar, GARFIELD-AF investigators

**Affiliations:** 1 Department of General Practice, Amsterdam UMC location University of Amsterdam, Amsterdam, Netherlands; 2 Thrombosis Research Institute, London, UK; 3 Amsterdam Public Health, Personalized Medicine, Amsterdam, Netherlands; 4 Cardiology, St George's Hospital, London, Ohio, USA; 5 Cardiology, Memorial Ankara Hospital, Ankara, Turkey; 6 University of the Witwatersrand Johannesburg, Johannesburg, South Africa; 7 Sir Ganga Ram Hospital, New Delhi, India; 8 Cardiology, National University Heart Centre, Singapore; 9 Medicine Alfred Hospital, Monash University, Melbourne, Victoria, Australia; 10 Tokai University School of Medicine Graduate School of Medicine, Isehara, Kanagawa, Japan; 11 Haemostasis and Thrombosis Research Group, Institute for Experimental Oncology and Therapy Research, Formerly Technical University Munich, Munich, Germany; 12 Cardiology, University of Edinburgh and Royal Infirmary, Edinburgh, UK; 13 University Hospital Motol, Prague, Czech Republic; 14 Cardiology, Onze Lieve Vrouwe Gasthuis, Amsterdam, Netherlands; 15 Department of Surgery, University College London, London, UK

**Keywords:** Atrial Fibrillation, Biostatistics, STROKE, Pharmacology, Clinical

## Abstract

**Background:**

The extent to which differences in results from Apixaban for Reduction in Stroke and Other Thromboembolic Events in Atrial Fibrillation (ARISTOTLE) and Rivaroxaban Once Daily Oral Direct Factor Xa Inhibition Compared with Vitamin K Antagonism for Prevention of Stroke and Embolism Trial (ROCKET) atrial fibrillation (AF)—the landmark trials for the approval of apixaban and rivaroxaban, respectively, for non-valvular AF—were influenced by differences in their protocols is debated. The potential influence of selection criteria on trial results was assessed by emulating these trials in data from the Global Anticoagulant Registry in the Field (GARFIELD)-AF registry.

**Methods:**

Vitamin K antagonist (VKA) and non-vitamin K oral antagonist (NOAC) users from GARFIELD-AF were selected according to eligibility for the original ARISTOTLE or ROCKET AF trials. A propensity score overlap weighted Cox model was used to emulate trial randomisation between treatment groups. Adjusted HRs for stroke or systemic embolism (SE) within 2 years of enrolment were calculated for each NOAC versus VKA.

**Results:**

Among patients on apixaban, rivaroxaban and VKA, 2570, 3560 and 8005 were eligible for ARISTOTLE, respectively, and 1612, 2005 and 4368, respectively, for ROCKET AF. When selecting for ARISTOTLE criteria, apixaban users had significantly lower stroke/SE risk versus VKA (HR 0.57; 95% CI 0.34 to 0.94) while no reduction was observed with rivaroxaban (HR 0.98; 95% CI 0.68 to 1.40). When selecting for ROCKET AF criteria, safety and efficacy versus VKA were similar across the NOACs.

**Conclusion:**

Apixaban and rivaroxaban showed similar results versus VKA in high-risk patients selected according to ROCKET AF criteria, whereas differences emerged when selecting for the more inclusive ARISTOTLE criteria. Our results highlight the importance of trial selection criteria in interpreting trial results and underline the problems faced in comparing treatments across rather than within clinical trials.

**WHAT IS ALREADY KNOWN ON THIS TOPIC**

- It has been suggested that differences in trial design contributed to outcomes of the landmark trials for the approval of apixaban (Apixaban for Reduction in Stroke and Other Thromboembolic Events in Atrial Fibrillation, ARISTOTLE) and rivaroxaban (Rivaroxaban Once Daily Oral Direct Factor Xa Inhibition Compared with Vitamin K Antagonism for Prevention of Stroke and Embolism Trial in Atrial Fibrillation (ROCKET AF)) for non-valvular atrial fibrillation. This has hampered comparison of the safety and efficacy of the two non-vitamin K oral anticoagulants based on the trial data.

**WHAT THIS STUDY ADDS**

- By emulating the randomised clinical trials in a real-world patient population, this study shows that all three oral anticoagulants achieved similar results in high-risk patients selected according to ROCKET AF criteria. In patients selected according to the more inclusive ARISTOTLE criteria, apixaban but not rivaroxaban showed clinical benefit compared with vitamin K antagonist for reducing the risk of stroke/systemic embolism.

**HOW THIS STUDY MIGHT AFFECT RESEARCH, PRACTICE OR POLICY**

- The results highlight the importance of trial selection criteria for interpreting trial results and underline the importance of high-quality observational data for assessment of relative drug performance in real-world populations.

## Introduction

 Oral anticoagulation (OAC) treatment effectively decreases the burden of stroke and mortality in at-risk atrial fibrillation (AF) patients. Non-vitamin K oral anticoagulants (NOACs) are non-inferior to vitamin K antagonists (VKA) in reducing stroke risk while being associated with a lower risk of bleeding.^1^ If indicated from the CHA_2_DS_2-_VASc score, current guidelines recommend NOACs rather than VKAs for stroke prophylaxis in non-valvular AF, without making general recommendations regarding specific NOACs.^1^,^3^ The most recent NICE guidance notes an absence of direct clinical head-to-head comparisons that would allow such a distinction.^3^

Researchers are, therefore, dependent on an indirect approach, comparing safety and efficacy outcomes of published randomised trials of various NOACs versus VKA. This approach, however, is hampered by considerably different study protocols.^4^ This applies specifically to the two landmark trials for approval of the factor Xa inhibitors apixaban and rivaroxaban—ARISTOTLE (Apixaban for Reduction in Stroke and Other Thromboembolic Events in AF) and ROCKET AF (Rivaroxaban Once Daily Oral Direct Factor Xa Inhibition Compared with Vitamin K Antagonism for Prevention of Stroke and Embolism Trial in AF).^5 6^ In these trials, non-identical patient selection criteria resulted in much higher cardiovascular disease burden among patients in ROCKET AF compared with ARISTOTLE. Moreover, these trials used different definitions for their primary safety endpoint, further impeding a comparison of the relative efficacy and safety of each NOAC versus VKA.

In the current work, we investigate the influence of the trials’ inclusion and exclusion criteria on results for safety and efficacy of apixaban and rivaroxaban versus VKA using uniform endpoints in an international AF patient registry.

## Methods

### Global Anticoagulant Registry in the Field-AF registry

The Global Anticoagulant Registry in the Field (GARFIELD-AF) registry is a prospective, multinational observational study that includes patients with newly diagnosed non-valvular AF from 1215 representative sites across 35 countries.^7^ Adults diagnosed with AF in the past 6 weeks, and at least one risk factor for stroke according to their local practitioner, were eligible for inclusion. Excluded were patients with a transient reversible cause for AF. Patients were enrolled prospectively and consecutively in five separate, sequential cohorts in order to minimise recruitment bias.

Choice of prophylactic treatment was at the discretion of the local practitioner. All participants were followed up for 2 years after enrolment. Inclusion and follow-up in GARFIELD-AF have been completed, and the database has been closed.

### Patient selection

The current analysis involved patients from GARFIELD-AF cohorts 3–5 (n=34 903, recruited from April 2013 to August 2016) who were treated at baseline with either apixaban, rivaroxaban or VKA. GARFIELD-AF cohort 1 (enrolment period: 2010–2011) and cohort 2 (2011–2013) were not included because NOACs had not been introduced in many participating countries at that time. Baseline treatment was defined as a participant’s first registered stroke prophylaxis by OAC, regardless of dosage or concomitant antiplatelet (AP) use. Patients were excluded if treatment or follow-up information was missing.

### Baseline data collection

Oversight of operations and data management of the GARFIELD-AF registry were conducted by the Thrombosis Research Institute (TRI; London, UK), with support from Quintiles (Durham, North Carolina, USA), The University of Birmingham Department of Primary Care Clinical Sciences (Birmingham, UK), Thrombosis Research Group-Brigham and Women’s Hospital (Boston, Massachusetts, USA) and AIXIAL (Paris, France). Baseline data were captured at enrolment using an electronic case report form (eCRF) designed by Dendrite Clinical Systems (Henley-on-Thames, UK). TRI oversaw the completeness and accuracy of the data, as well as data queries to study sites. A 20% portion of all eCRFs were monitored against source documentation. An electronic audit trail existed for all data modifications, and critical variables were subjected to further audits.^7 8^ Data for components of the CHADS_2_, CHA_2_DS_2_-VASc, HAS-BLED (Hypertension, Abnormal Renal/Liver Function, Stroke, Bleeding History or Predisposition, Labile INR, Elderly, Drugs/Alcohol Concomitantly) and GARFIELD-AF risk stratification schemes were collected and calculated retrospectively.^9^,^12^ Fluctuations in international normalised ratio were excluded from HAS-BLED score calculations. Follow-up data were collected at 4-month intervals up to 24 months. Data for this report were extracted from the study database on 30 June 2019.

Chronic kidney disease was classified according to National Kidney Foundation guidelines: moderate-to-severe (stages 3–5), mild (stages 1 and 2) or none. Ethnicity was classified by the investigator in agreement with the patient. Vascular disease was defined as peripheral artery disease and/or coronary artery disease. The CHADS_2_ scores were calculated using element definitions as applied in the respective original ARISTOTLE and ROCKET AF trials. The GARFIELD-AF risk scores for 2-year risk of mortality, non-haemorrhagic stroke/SE and major bleeding in newly diagnosed non-valvular AF patients were derived using their originally reported coefficients.^13^

### Outcomes

The primary efficacy outcome was the composite of stroke (haemorrhagic, ischaemic or unknown type) and systemic embolism (SE). The secondary efficacy outcome was all-cause mortality. The primary safety outcome was major bleeding characterised by one or more of the following symptoms as defined by the Scientific and Standardisation Committee of the International Society on Thrombosis and Haemostasis: clinically overt bleeding associated with a fall in haemoglobin of ≥2 g/dL, associated with transfusion of packed red blood cells or whole blood, bleeding with fatal outcome or bleeding in a critical site—namely intracranial (spontaneous intracerebral, intraventricular, subarachnoidal, subdural, epidural), intraspinal, pericardial, intra-articular, intramuscular with compartment syndrome, or retroperitoneal.^14^ Clinical events were defined prior to patient enrolment.^7^

### Landmark trial eligibility

Review of the protocols for ARISTOTLE and ROCKET AF identified 8 distinct inclusion and 49 exclusion criteria across both trials (online supplemental table S1).^5 6^ All of the inclusion criteria in both trials, and many of their exclusion criteria, could be matched with GARFIELD-AF eCRFs or were operationalised where possible as described in the table footnotes. GARFIELD-AF patients were considered eligible for ARISTOTLE or ROCKET AF if one or more trial inclusion criteria and no exclusion criteria were present at baseline.

### Statistical analysis

Frequencies and percentages are reported to summarise categorical variables, and medians and IQR for continuous variables.

Unadjusted event rates for outcomes of interest were calculated using Poisson regression and presented per 100 person-years with corresponding 95% CIs. Propensity scores of apixaban versus VKA and of rivaroxaban versus VKA were generated using logistic regression. Included confounders are shown in online supplemental table S2. These scores were applied as overlap weights to Cox proportional hazards models to obtain the adjusted HRs with 95% CIs for each NOAC versus VKA comparison, selecting for ARISTOTLE and ROCKET AF criteria.^15 16^ Time-to-event analyses included patients from time of enrolment until the outcome of interest, loss to follow-up or 2 years of follow-up, whichever came first.

Treatment was defined as the first treatment received at the time of enrolment, approximating ‘intention-to-treat’. Only cases with complete data for each covariate were presented in descriptive tables. The percentages of missing data per variable are shown in online supplemental table S3. Multiple imputation was applied for the comparative effectiveness analyses. Values missing from the patients’ baseline covariates were imputed with multivariate imputation by chained equations. SEs were obtained by combining estimates across five imputed databases. Data analysis was carried out using SAS Enterprise Guide vV.8.2 (SAS InstituteA).

## Results

**Figure 1:**
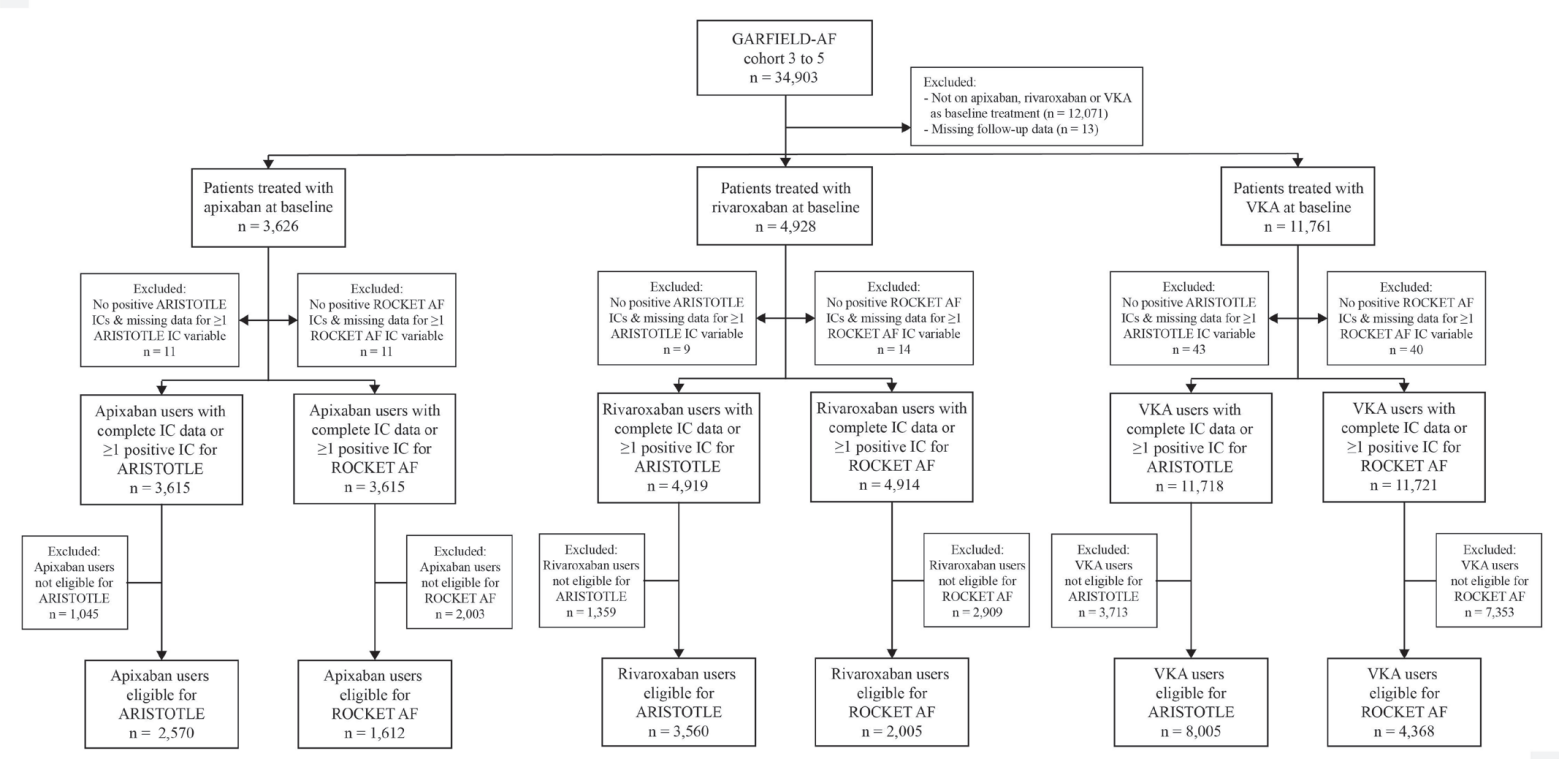
Study flow chart. ARISTOTLE, Apixaban for Reduction in Stroke and Other Thromboembolic Events in Atrial Fibrillation; GARFIELD-AF, Global Anticoagulant Registry in the FIELD; IC, inclusion criterion; ROCKET AF, Rivaroxaban Once Daily Oral Direct Factor Xa Inhibition Compared with Vitamin K Antagonism for Prevention of Stroke and Embolism Trial in Atrial Fibrillation; VKA, vitamin K antagonists.

All inclusion criteria and a number of key exclusion criteria of both trials were matched. Among the trial exclusion criteria that could not be verified from GARFIELD-AF data, a considerable number were either already excluded from GARFIELD-AF (eg, reversible cause for AF) or clinically unlikely to have been incorporated in a database of real-world newly diagnosed AF patients treated with OAC (eg, planned or recent major surgery, or active or recent major bleeding—see online supplemental table S1 and footnotes). Among GARFIELD-AF patients who met the trial inclusion and exclusion criteria for ARISTOTLE, 2570 were taking apixaban, 3560 rivaroxaban and 8005 VKAs. For ROCKET AF, the equivalent patient numbers were 1612, 2005 and 4368, respectively (figure 1).

Table 1 shows the baseline characteristics of OAC users eligible for ARISTOTLE. Apixaban users were older than those on rivaroxaban or VKA (median age 76, 73 and 71 years, respectively). Apixaban users also had the highest median CHA_2_DS_2_-VASc score (4.0 vs 3.0 for rivaroxaban and VKA), and the largest proportion of patients with a CHADS_2_ score ≥3 (28.7%, 24.4% and 25.5%, respectively). HAS-BLED scores were similarly distributed among all groups. The 2-year risks for mortality, stroke and bleeding as per the GARFIELD-AF scores were highest in VKA users, followed by participants on apixaban and rivaroxaban.

**Table 1:** Baseline characteristics for ARISTOTLE trial eligible patients by OAC treatment at baseline

		Treatment at baseline	
Baseline characteristics	Apixaban (n=2570)	Rivaroxaban (N=3560)	VKA (N=8005)
Female sex, n (%)	1240 (48.2)	1599 (44.9)	3747 (46.8)
Age, median (Q1; Q3), years	76.0 (69.0; 81.0)	73.0 (65.0; 79.0)	71.0 (64.0; 77.0)
Ethnicity, n (%)			
Caucasian	1513 (60.8)	2507 (71.5)	5296 (67.3)
Hispanic/Latino	70 (2.8)	199 (5.7)	666 (8.5)
Asian	859 (34.5)	722 (20.6)	1730 (22.0)
Afro-Caribbean/mixed/other	46 (1.8)	79 (2.3)	174 (2.2)
Body mass index, median (Q1; Q3), kg/m²	26.2 (23.4; 29.8)	27.5 (24.4; 31.3)	27.8 (24.6; 32.0)
Systolic blood pressure, median (Q1; Q3), mm Hg	130.0 (120.0; 142.0)	132.0 (120.0; 144.0)	130.0 (120.0; 144.0)
Diastolic blood pressure, median (Q1; Q3), mm Hg	79.0 (70.0; 85.0)	80.0 (70.0; 86.0)	80.0 (70.0; 88.0)
Pulse, median (Q1; Q3), bpm	84.0 (70.0; 107.0)	84.0 (70.0; 105.0)	84.0 (72.0; 100.0)
Type of atrial fibrillation, n (%)			
Permanent	340 (13.2)	472 (13.3)	1398 (17.5)
Persistent	392 (15.3)	587 (16.5)	1358 (17.0)
Paroxysmal	895 (34.8)	1104 (31.0)	1736 (21.7)
New onset (unclassified)	943 (36.7)	1397 (39.2)	3513 (43.9)
Care setting specialty at diagnosis, n (%)			
Internal medicine/neurology/geriatrics	486 (18.9)	679 (19.1)	1558 (19.5)
Cardiology	1826 (71.1)	2439 (68.5)	5122 (64.0)
Primary care/general practice	258 (10.0)	442 (12.4)	1325 (16.6)
Care setting location at diagnosis, n (%)			
Hospital	1190 (46.3)	1653 (46.4)	4643 (58.0)
Office/anticoagulation clinic/thrombosis centre	1142 (44.4)	1582 (44.4)	2514 (31.4)
Emergency room	238 (9.3)	325 (9.1)	848 (10.6)
Medical history, n (%)			
Heart failure	567 (22.1)	820 (23.0)	2046 (25.6)
Acute coronary syndromes	227 (8.9)	336 (9.5)	798 (10.0)
Vascular disease*	491 (19.3)	802 (22.8)	1947 (24.6)
Carotid occlusive disease	90 (3.6)	97 (2.8)	176 (2.2)
VTE	53 (2.1)	108 (3.1)	260 (3.3)
Prior stroke/TIA/SE	366 (14.4)	375 (10.6)	1032 (13.0)
Hypertension	2082 (81.2)	3020 (84.9)	6761 (84.7)
Hypercholesterolaemia	1114 (44.3)	1655 (48.2)	3506 (46.0)
Diabetes	621 (24.2)	917 (25.8)	2219 (27.7)
Moderate to severe CKD	219 (8.8)	288 (8.4)	469 (6.3)
Current smoker, n (%)	181 (7.9)	309 (9.6)	694 (9.4)
AP treatment, n (%)	372 (14.7)	539 (15.4)	1665 (20.8)
CHADS2 score, median (Q1; Q3)	2.0 (1.0; 3.0)	2.0 (1.0; 2.0)	2.0 (1.0; 3.0)
CHADS2 score 0–1, n (%)	707 (29.4)	1124 (33.7)	2345 (31.5)
CHADS2 score 2, n (%)	1005 (41.8)	1398 (41.9)	3198 (43.0)
CHADS2 score≥3, n (%)	690 (28.7)	815 (24.4)	1896 (25.5)
CHA2DS2-VASc score, median (Q1; Q3)	4.0 (3.0; 4.0)	3.0 (2.0; 4.0)	3.0 (3.0; 4.0)
HAS-BLED score†, median (Q1; Q3)	1.0 (1.0; 2.0)	1.0 (1.0; 2.0)	1.0 (1.0; 2.0)
GARFIELD-AF mortality score§‡, median (Q1; Q3)	4.6 (2.9; 7.7)	4.1 (2.4; 6.9)	5.0 (3.0; 7.7)
GARFIELD-AF stroke score §¶, median (Q1; Q3)	1.4 (1.0; 1.9)	1.2 (0.9; 1.7)	1.5 (1.1; 2.0)
GARFIELD-AF bleeding score§**, median (Q1; Q3)	1.7 (1.2; 2.2)	1.5 (1.1; 2.0)	2.1 (1.5; 2.7)

* Defined as peripheral artery disease and/or coronary artery disease.

† The risk factor ‘labile INRs’ is not included in the HAS-BLED score as it is not collected at baseline. As a result, the maximum HAS-BLED score at baseline is 8 points (not 9).

‡ Represents the expected probability of dying within 2 years follow-up.

§ The GARFIELD-AF risk scores use different coefficients for NOAC and VKA treatment, leading to higher estimated risk in VKA users versus NOAC users with the same baseline characteristics. The scores are not impacted by the type of NOAC.

¶ Represents the expected probability of developing a non-haemorrhagic stroke/SE within 2 years follow-up.

** Represents the expected probability of developing a major bleeding within 2 years follow-up.

AP, antiplatelet; ARISTOTLE, Apixaban for Reduction in Stroke and Other Thromboembolic Events; CKD, chronic kidney disease; GARFIELD, Global Anticoagulant Registry in the Field; HAS-BLED, Hypertension, Abnormal Renal/Liver Function, Stroke, Bleeding History or Predisposition, Labile INR, Elderly, Drugs/Alcohol Concomitantly; NOAC, non-vitamin K oral antagonist; OAC, oral anticoagulation; SE, systemic embolism; TIA, transient ischaemic attack; VKA, vitamin K antagonist; VTE, venous thromboembolism.

The median age among ROCKET AF-eligible apixaban, rivaroxaban and VKA users followed the same trend (78 vs 76 and 75 years, respectively) as seen above for patients selected according to ARISTOTLE criteria (table 2). Median CHADS_2_, CHA_2_DS_2_-VASc and HAS-BLED scores were similar between all OAC groups. However, the proportion of patients with a CHADS_2_ score ≥3 was lower among those on VKA than in those on apixaban or rivaroxaban (37.0% vs 42.6% and 39.3%, respectively). As in the ARISTOTLE-eligible patients above, median GARFIELD-AF risk scores for mortality, stroke and bleeding were numerically higher for VKA than for apixaban or rivaroxaban users.

**Table 2:** Baseline characteristics for ROCKET AF trial eligible patients by OAC treatment at baseline

		Treatment at baseline	
Baseline characteristics	Apixaban (n=1612)	Rivaroxaban (N=2005)	VKA (N=4368)
Female sex, n (%)	793 (49.2)	949 (47.3)	2072 (47.4)
Age, median (Q1; Q3), years	78.0 (73.0; 82.0)	76.0 (70.0; 81.0)	75.0 (66.0; 79.0)
Ethnicity, n (%)			
Caucasian	899 (57.2)	1389 (70.5)	2930 (68.3)
Hispanic/Latino	50 (3.2)	137 (7.0)	405 (9.4)
Asian	605 (38.5)	413 (21.0)	862 (20.1)
Afro-Caribbean/mixed/other	19 (1.2)	31 (1.6)	94 (2.2)
Body mass index, median (Q1; Q3), kg/m²	25.7 (23.1; 29.3)	27.5 (24.2; 31.2)	27.6 (24.5; 31.7)
Systolic blood pressure, median (Q1; Q3), mm Hg	130.0 (120.0; 141.0)	132.0 (120.0; 142.0)	130.0 (120.0; 142.0)
Diastolic blood pressure, median (Q1; Q3), mm Hg	78.0 (70.0; 83.0)	80.0 (70.0; 85.0)	80.0 (70.0; 85.0)
Pulse, median (Q1; Q3), bpm	82.0 (70.0; 100.0)	82.0 (70.0; 100.0)	83.0 (71.0; 100.0)
Type of atrial fibrillation, n (%)			
Permanent	254 (15.8)	327 (16.3)	878 (20.1)
Persistent	250 (15.5)	294 (14.7)	669 (15.3)
Paroxysmal	580 (36.0)	602 (30.0)	891 (20.4)
New onset (unclassified)	528 (32.8)	782 (39.0)	1930 (44.2)
Care setting specialty at diagnosis, n (%)			
Internal medicine/neurology/geriatrics	351 (21.8)	438 (21.8)	895 (20.5)
Cardiology	1123 (69.7)	1331 (66.4)	2694 (61.7)
Primary care/general practice	138 (8.6)	236 (11.8)	779 (17.8)
Care setting location at diagnosis, n (%)			
Hospital	705 (43.7)	918 (45.8)	2518 (57.6)
Office/anticoagulation clinic/thrombosis centre	781 (48.4)	926 (46.2)	1419 (32.5)
Emergency room	126 (7.8)	161 (8.0)	431 (9.9)
Medical history, n (%)			
Heart failure	423 (26.2)	594 (29.6)	1353 (31.0)
Acute coronary syndromes	156 (9.7)	211 (10.6)	486 (11.2)
Vascular disease*	339 (21.3)	512 (25.8)	1153 (26.7)
Carotid occlusive disease	65 (4.1)	60 (3.1)	110 (2.6)
VTE	30 (1.9)	54 (2.7)	117 (2.7)
Prior stroke/TIA/SE	329 (20.6)	324 (16.3)	758 (17.5)
Hypertension	1338 (83.2)	1715 (85.6)	3698 (84.9)
Hypercholesterolaemia	712 (44.9)	955 (49.4)	1993 (47.8)
Diabetes	495 (30.7)	712 (35.5)	1629 (37.3)
Moderate to severe CKD	161 (10.4)	201 (10.4)	293 (7.2)
Current smoker, n (%)	88 (6.1)	154 (8.5)	360 (9.0)
AP treatment, n (%)	251 (15.8)	328 (16.7)	994 (22.8)
CHADS2 score, median (Q1; Q3)	2.0 (2.0; 3.0)	2.0 (2.0; 3.0)	2.0 (2.0; 3.0)
CHADS2 score 0–1, n (%)	0 (0.0)	0 (0.0)	0 (0.0)
CHADS2 score 2, n (%)	915 (57.4)	1208 (60.7)	2726 (63.0)
CHADS2 score≥3, n (%)	679 (42.6)	783 (39.3)	1600 (37.0)
CHA2DS2-VASc score, median (Q1; Q3)	4.0 (3.0; 5.0)	4.0 (3.0; 5.0)	4.0 (3.0; 5.0)
HAS-BLED score†, median (Q1; Q3)	1.0 (1.0; 2.0)	1.0 (1.0; 2.0)	1.0 (1.0; 2.0)
GARFIELD-AF mortality score‡§, median (Q1; Q3)	5.6 (3.7; 8.8)	5.6 (3.6; 8.5)	6.0 (4.0; 8.8)
GARFIELD-AF stroke score§¶, median (Q1; Q3)	1.5 (1.2; 2.1)	1.5 (1.2; 1.9)	1.7 (1.3; 2.1)
GARFIELD-AF bleeding score§**, median (Q1; Q3)	1.9 (1.4; 2.4)	1.8 (1.4; 2.3)	2.3 (1.7; 2.9)

* Defined as peripheral artery disease and/or coronary artery disease.

† The risk factor ‘labile INRs’ is not included in the HAS-BLED score as it is not collected at baseline. As a result, the maximum HAS-BLED score at baseline is 8 points (not 9).

‡ Represents the expected probability of dying within 2 years follow-up.

§ The GARFIELD-AF risk scores use different coefficients for NOAC and VKA treatment, leading to higher estimated risk in VKA users versus NOAC users with the same baseline characteristics. The scores are not impacted by the type of NOAC.

¶ Represents the expected probability of developing a non-haemorrhagic stroke/SE within 2 years follow-up.

** Represents the expected probability of developing a major bleeding within 2 years follow-up.

AP, antiplatelet; CKD, chronic kidney disease; GARFIELD, Global Anticoagulant Registry in the Field; HAS-BLED, Hypertension, Abnormal Renal/Liver Function, Stroke, Bleeding History or Predisposition, Labile INR, Elderly, Drugs/Alcohol Concomitantly; INR, international normalised ratio; NOAC, non-vitamin K oral antagonist; ROCKET, Rivaroxaban Once Daily Oral Direct Factor Xa Inhibition Compared with Vitamin K Antagonism for Prevention of Stroke and Embolism Trial; SE, systemic embolism; TIA, transient ischaemic attack; VKA, vitamin K antagonist; VTE, venous thromboembolism.

### Comparative effectiveness analyses

**Figure 2:**
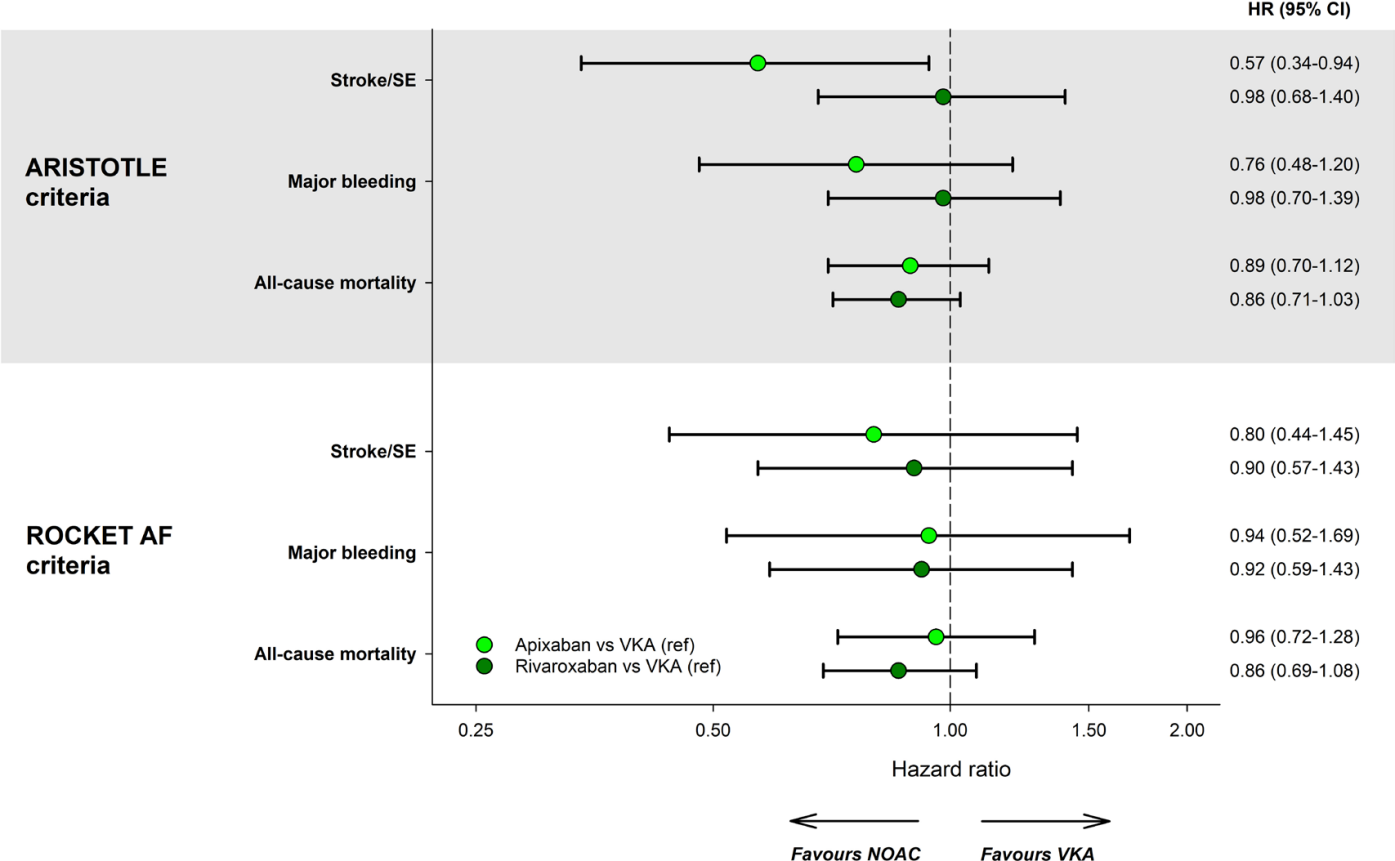
Propensity-score weighted HRs comparing apixaban or rivaroxaban versus VKA as reference. Shown are HRs for selected outcomes through 2 years of follow-up in the study populations selected using ARISTOTLE and ROCKET AF criteria. HR obtained using an overlap-weighted Cox model that included the following variables in the weighting scheme: country and cohort enrolment, sex, age, ethnicity, type of AF, care setting specialty and location, congestive heart failure, acute coronary syndromes, vascular disease, carotid occlusive disease, prior stroke/transient ischaemic attack/SE, prior bleeding, VTE, hypertension, hypercholesterolaemia, diabetes, moderate to severe chronic kidney disease, hyperthyroidism, hypothyroidism, current smoking, heavy alcohol consumption, body mass index, heart rate, systolic and diastolic blood pressure at diagnosis, and baseline antiplatelet use. AF, atrial fibrillation; ARISTOTLE, Apixaban for Reduction in Stroke and Other Thromboembolic Events; NOAC, non-vitamin K antagonist; ROCKET, Rivaroxaban Once Daily Oral Direct Factor Xa Inhibition Compared with Vitamin K Antagonism for Prevention of Stroke and Embolism Trial; SE, systemic embolism; VKA, vitamin K antagonist; VTE, venous thromboembolism.

VKA users had the highest crude event rates per 100 person-years for all outcomes in ARISTOTLE as well as ROCKET AF-based analyses. In addition, event rates were generally lower in patients on apixaban compared with rivaroxaban, regardless of trial criteria (table 3). However, after propensity score weighting, there were no substantial differences regarding the effectiveness of NOAC versus VKA, or their relative effectiveness in patients selected according to ARISTOTLE versus ROCKET AF criteria (figure 2). However, the use of apixaban was associated with a significantly reduced risk of stroke/SE compared with VKA in ARISTOTLE-eligible participants (HR 0.57; 95% CI 0.34 to 0.94) while no reduction was observed with rivaroxaban (HR 0.98; 95% CI 0.68 to 1.40). When selecting for ROCKET AF criteria, no significant associations were observed, with safety and efficacy versus VKA being similar between the two NOACs. All confounders in the propensity score were balanced between NOAC and VKA after weighting (online supplemental figures S1–S4), indicating that the emulated ‘treatment arms’ were comparable for all considered confounders after the weighting scheme was applied.

**Table 3:** Crude event rates (per 100 person-years) within 2-year follow-up for patients eligible for ARISTOTLE and ROCKET AF by baseline OAC treatment

		Apixaban			Rivaroxaban			VKA		
	Outcome	N total	N events	Rate (95% CI)	N total	N events	Rate (95% CI)	N total	N events	Rate (95% CI)
	Stroke/SE	2570	32	0.7 (0.5 to 0.9)	3560	57	0.8 (0.6 to 1.1)	8005	152	1.0 (0.9 to 1.2)
ARISTOTLE	Major bleeding	2570	40	0.8 (0.6 to 1.1)	3560	66	1.0 (0.8 to 1.2)	8005	165	1.1 (0.9 to 1.3)
Criteria	All-cause mortality	2570	151	3.0 (2.6 to 3.6)	3560	208	3.0 (2.7 to 3.5)	8005	566	3.8 (3.5 to 4.1)
	Stroke/SE	1612	28	0.9 (0.6 to 1.3)	2005	39	1.0 (0.8 to 1.4)	4368	90	1.1 (0.9 to 1.4)
ROCKET AF	Major bleeding	1612	27	0.9 (0.6 to 1.3)	2005	44	1.2 (0.9 to 1.6)	4368	98	1.2 (1.0 to 1.5)
Criteria	All-cause mortality	1612	119	3.9 (3.2 to 4.6)	2005	158	4.2 (3.6 to 4.9)	4368	371	4.5 (4.1 to 5.0)

ARISTOTLE, Apixaban for Reduction in Stroke and Other Thromboembolic Events in Atrial Fibrillation; OAC, oral anticoagulation; ROCKET AF, Rivaroxaban Once Daily Oral Direct Factor Xa Inhibition Compared with Vitamin K Antagonism for Prevention of Stroke and Embolism Trial in Atrial Fibrillation; SE, systemic embolism; VKA, vitamin K antagonist.

## Discussion

We used a novel approach to assess the potential influence of trial inclusion criteria on reported safety and efficacy by emulating two landmark NOAC versus VKA trials in real-world AF patients. Patient baseline characteristics and 2-year follow-up matched conditions of the ARISTOTLE and ROCKET AF landmark studies.^5 6^ The main finding is that the relative effectiveness of all three OACs was similar when applying the ROCKET AF criteria, and thus selecting patients at a higher risk of stroke. By contrast, when the more inclusive ARISTOTLE criteria were applied, apixaban, and to a lesser extent rivaroxaban, demonstrated significant clinical benefits in terms of stroke/SE risk compared with VKA. These findings are not intended to guide the prescription of specific OACs in stroke prophylaxis, rather they highlight the importance of selection criteria when designing and interpreting clinical trials.

The adjusted HRs in observational data showed considerable overlap with the results of the original trials in all selected outcomes. Differences in the risk of selected outcomes between ROCKET AF and our emulation of the trial could be due to the fact that the trial design limited the number of recruited low-risk patients. We did not apply the same restrictions to the GARFIELD-AF dataset because doing so would have further reduced the number of eligible rivaroxaban users, and thereby the statistical power in our analysis.

Interestingly, applying the ARISTOTLE selection criteria to the GARFIELD-AF registry did not replicate the original trial’s finding that apixaban reduces major bleeding compared with VKA.^5^ This discrepancy might be due to differences in the proportion of patients with moderate-to-severe renal impairment. In our cohort, 8.8% of apixaban-treated and 6.3% of VKA-treated patients had moderate-to-severe renal impairment, compared with approximately 16.5% in both arms of ARISTOTLE. Subgroup analysis of ARISTOTLE showed apixaban’s protection against major bleeding varied according to renal impairment level, benefiting those with moderate-to-severe impairment but not those with no impairment. Therefore, the lower proportion of patients with significant renal impairment in our study could partially explain the lack of association between apixaban and major bleeding.

Randomised clinical trials remain the gold standard of medical research. However, results of NOAC versus VKA trials are difficult to compare due to markedly different baseline characteristics of the participants, as outlined by numerous reports.^417^,^20^ When direct comparisons through randomised trials are unavailable, high-quality observational data are required for assessing relative drug performance.^21 22^ The GARFIELD-AF registry contains extensive data for baseline characteristics and outcomes over a 2-year follow-up period, similar to the original ARISTOTLE and ROCKET AF trials. This enabled a valid emulation of both trials’ inclusion and exclusion criteria.

Several studies suggest that NOACs might have different risk/benefit profiles in AF patients, especially when not at a high risk of stroke. The largest and most comprehensive observational study comparing NOACs in patients with AF found that apixaban was associated with the lowest risk for gastrointestinal bleeding among all NOACs, but similar rates of ischaemic stroke or SE and intracranial haemorrhage. Estimated risks among users of apixaban versus rivaroxaban for gastrointestinal bleeding (HR 0.72, 95% CI 0.66 to 0.79) and ischaemic stroke/SE (HR 0.89, 95% CI 0.78 to 1.02) were in line with earlier large observational studies and meta-analysis.^23^ Ray *et al*, with a larger number of patients on apixaban and rivaroxaban, provided more precise estimates for intracranial haemorrhage (HR 0.68, 95% CI 0.59 to 0.77) and all-cause mortality (HR 0.94, 95% CI 0.92 to 0.98).^21^ A method for indirectly comparing NOACs is network meta-analysis of randomised controlled trials. A systematic review of 22 such studies concluded that no significant difference existed between apixaban and rivaroxaban regarding the risk for stroke/SE, but that apixaban had a lower risk for major bleeding.^24^

As mentioned above, these results need to be interpreted cautiously due to differences between the designs of the original trials.^4^ Interestingly, one meta-analysis observed that among very high-risk patients in ARISTOTLE and ROCKET AF (CHADS_2_ score ≥3) the risks of stroke or death were similar, irrespective of the NOAC used. However, unlike in our ROCKET AF selected patients, significantly fewer major haemorrhage events occurred in the high-risk apixaban compared with rivaroxaban users.^25^

The GARFIELD-AF registry’s active enrolment coincided with the emergence of NOACs for use in non-valvular AF. Previous work in the registry confirmed safety and efficacy of NOACs versus VKA overall, and in subgroup populations of newly diagnosed AF patients.^26 27^

### Strengths and limitations

The main strengths of this analysis arise from the GARFIELD-AF registry as the largest worldwide prospective observational cohort of newly diagnosed AF patients. A detailed and highly complete follow-up allowed for the assessment of 2-year outcomes and emulation of two randomised trials in the same dataset. Studies with smaller sample sizes or shorter follow-up can be insufficiently powered to detect differences in the rates of rare events between treatment groups.

The extensive baseline investigation enabled assessment of all inclusion, as well as the main exclusion criteria of the ARISTOTLE and ROCKET AF trials. It also allowed for a detailed presentation of baseline characteristics, which showed the effect of different eligibility criteria on comorbidity and risk profiles of eligible participants. Further strengths are the use of propensity score overlap weights to ensure comparisons of NOAC versus VKA in patients with similar baseline properties, and use of similar outcome definitions to compare results, unlike the different definitions used in the original trials.^4^,^6^

This work had several limitations. Treatment was defined as first OAC received, analogous to an intention-to-treat analysis, not accounting for non-recommended dosing, treatment switches or cessation. Not all exclusion criteria defined in the original ARISTOTLE and ROCKET AF protocols could be operationalised in the GARFIELD-AF dataset. However, the main reasons for non-operationalised trial exclusion were clinically established contraindications to any OAC, for example, planned major surgery or recent stroke or major bleeding. Therefore, it was unlikely that such patients were enrolled in GARFIELD-AF and thus wrongfully included in the current analysis.

The geographical catchment of the trials differed from that of GARFIELD-AF which covered over 30 countries. We previously observed geographical variation in the outcomes of OAC treatment in AF patients that was not explained by baseline risk factors and likely due to regional differences in clinical practice, including the management of comorbidities.^28^ Patients of black African ancestry were likely underrepresented in all of the studies.

Due to the more stringent selection criteria for ROCKET AF, this trial’s emulation contained fewer patients, resulting in wider CIs and less certainty in the observed trends. Unlike ROCKET AF, we did not restrict the proportion of patients with CHADS_2_ score ≤2 to a maximum of 10% of the sample. Consequently, the overall cardiovascular risk in the population of the emulated trial was lower than in the original trial.

Fourth, we did not include GARFIELD-AF participants on dabigatran or edoxaban at baseline. The registry contained few edoxaban users because this NOAC had not yet been widely introduced during GARFIELD-AF enrolment. As for dabigatran, its landmark trial reported separate results for the doses 150 and 110 mg.^29^ However, the GARFIELD-AF registry contained too few dabigatran users to allow for stratification into separate dabigatran dosage arms in our analyses.

Finally, although we adjusted for an extensive list of confounding factors, in an observational study, the possibility of unmeasured confounding cannot be ruled out. Therefore, definite conclusions regarding superiority of any NOAC will require direct comparison in carefully designed randomised trials.

## Conclusion

Apixaban and rivaroxaban had similar results versus VKA when emulating trials in higher-risk patients using ROCKET AF criteria. When the more inclusive ARISTOTLE criteria were applied, apixaban demonstrated clinical benefits compared with VKA in terms of reducing the risk of stroke/SE. These observations provide insights into the role of trial inclusion criteria in trial result interpretation and underline the importance of high-quality observational data for assessment of relative drug performance in populations who are treated according to local practices and not in the confines of a randomised trial. Such randomised trials will be needed to inform policy decisions for clinicians who wish to initiate an NOAC in their recently diagnosed AF patients.

## Supplementary material

10.1136/openhrt-2024-002708 online supplemental file 1

## Data Availability

Requests for patient level data can be made to SV, head of statistics at the Thrombosis Research Institute (svirdone@tri-london.ac.uk). These requests should include a protocol summary and a summary of the statistical analysis plan. The request will be reviewed by the data sharing committee for approval and next steps will be discussed with the requestor.
